# Recent Advances in Silica-Nanomaterial-Assisted Lateral Flow Assay

**DOI:** 10.3390/bioengineering9070266

**Published:** 2022-06-21

**Authors:** Han Zhuang, Chun Xu, Fang Gao, Yiwei Li, Chang Lei, Chengzhong Yu

**Affiliations:** 1 Australian Institute for Bioengineering and Nanotechnology, The University of Queensland, St Lucia, QLD 4072, Australia; han.zhuang@uq.net.au (H.Z.); f.gao@uq.net.au (F.G.); yiwei.li@uq.net.au (Y.L.); 2 School of Dentistry, The University of Queensland, Brisbane, QLD 4006, Australia; chun.xu@uq.edu.au

**Keywords:** lateral flow assay, point-of-care device, silica nanomaterials, signal amplification

## Abstract

Lateral flow assays (LFAs) have attracted much attention as rapid and affordable point-of-care devices for medical diagnostics. The global SARS-CoV-2 pandemic has further highlighted the importance of LFAs. Many efforts have been made to enhance the sensitivity of LFAs. In recent years, silica nanomaterials have been used to either amplify the signal of label materials or provide stability, resulting in better detection performance. In this review, the recent progress of silica-nanomaterial-assisted LFAs is summarized. The impact of the structure of silica nanomaterials on LFA performance, the challenges and prospects in this research area are also discussed.

## 1. Introduction

Lateral flow assays (LFAs), also known as lateral flow tests, are point-of-care tools designed to detect the presence of target analytes in samples. First emerging on the commercial market in the 1970s, LFAs were used to detect human chorionic gonadotropin (hCG) in urine in order to test for pregnancy [1]. Since then, LFAs have been widely used for various biological diagnoses, and their global market size is projected to reach USD 11.7 billion by 2028 [2]. In 2019, the global SARS-CoV-2 pandemic further highlighted the importance of LFAs. Currently, there are 50 SARS-CoV-2 self-testing LFA kits approved by the FDA under emergency use authorization [3]. Self-testing LFA kits enable a timely diagnosis of infected people, even those without any symptoms, thus suppressing the spread of the virus and significantly enhancing the capacity to detect COVID-19.

**Figure 1:**
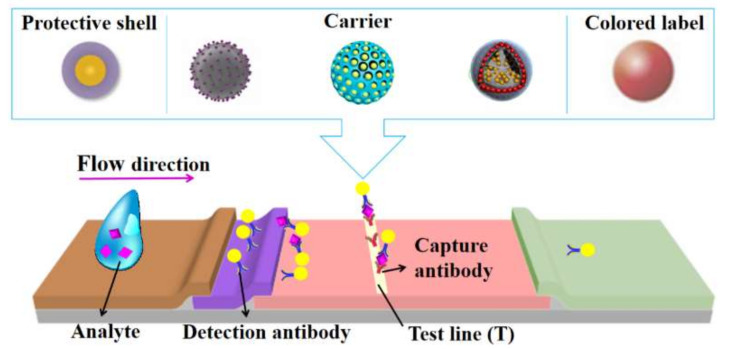
Schematic illustration of a silica-nanomaterial-assisted lateral flow assay (LFA). In LFAs, silica nanomaterials usually work as a protective shell to stabilize label materials, a carrier to enrich label materials for signal amplification or the colored label itself. Reprinted with permission from References [20,21,22,23].

LFAs have been used to detect a wide range of analytes, such as hormones [4], pesticide residues [5], microorganisms [6], antibodies [7] and drugs [8], in a variety of samples, including blood, saliva, urine, nasal fluid and other fluids. The general working principle of LFAs is based on the movement of a liquid sample across a strip via capillary force and antigen–antibody interactions. Typically, a standard LFA strip has four parts: a sample pad (the sample dropping area), a conjugate pad (the binding area of labeled tags and biorecognition elements), a membrane (usually a nitrocellulose membrane, which contains a test line and a control line) and an absorption pad to reserve waste liquid (Figure 1) [9]. Several LFA formats have been developed for the detection of different types of target analytes, including sandwich, competitive and multiplex detective formats. The sandwich format is the most typical one, in which the target analyte is sandwiched between a capture antibody and a detection antibody (Figure 1). This format is generally applied to detect large biomolecules. The competitive format is usually used to detect low-molecular-weight compounds, which are unable to bind two antibodies simultaneously [10]. Generally, this format has two arrangements. In the first arrangement, the target antigen competes with a reference antigen to bind to the labeled antibody. In the other arrangement, the target antigen competes with the labeled antigen to bind to the antibody. In both arrangements, a positive result will not show a signal at the test line, as labels cannot bind at the test line. The multiplex detective format is achieved by adding multiple test lines on a strip. It is designed for the detection of more than one target analyte at the same time [11].

Although LFAs have achieved great commercial success, they still suffer from a relatively low detection sensitivity [12]. To address this limitation, many efforts have been made to either develop label materials with a high signal intensity [13], fabricate a nanocomplex that enriches a large amount of label materials [14] or use catalysts to amplify the signal [15]. The development of LFA readers with improved resolution is also an important research direction [16,17,18]. To date, many materials, such as polymers and silica, have been applied in the above-mentioned strategies. Polymers are a big family of large molecules that have been widely used in biomedical applications. Despite its advantages, the framework stability of polymers is usually less robust than that of silica. Moreover, silica is better than polymers in providing large specific surface areas, adjustable pore sizes and high pore volumes for the enrichment of label materials [19]. Therefore, in this review, we focus on the use of silica nanomaterials to improve the performance of LFAs.

Silica nanomaterials are a big family of materials that have been widely used in various biomedical applications, including LFAs. The main role of silica nanomaterials is to work as label carriers in order to amplify the signal intensity and enhance the detection sensitivity of LFAs (Figure 1). Porous silica nanomaterials have large specific surface areas and tunable pore structures, which enable the enrichment of label materials for signal amplification. The pore size of silica nanomaterials can also be adjusted to accommodate label materials of different sizes. Moreover, the ease of the surface modification of silica nanomaterials facilitates the immobilization of label materials [24] and the further linking of antibodies. In addition to label carriers, silica nanomaterials also contribute to stabilizing label materials due to their stable nature.

In recent years, many studies reported the involvement of silica nanomaterials in LFAs with superior performance. The published reviews in this field are either summarized from a general material perspective [25,26] or only focused on a certain format of detection [27,28]. In this review, we focus on silica-material-assisted LFAs and discuss the recent research according to the signal formats, including colorimetric LFAs, fluorescent LFAs, chemiluminescent LFAs, electrochemical LFAs and surface-enhanced Raman scattering (SERS) LFAs. This review aims to present a selection of important research findings of LFAs from a new perspective, that is, to determine how the structural parameters (e.g., diameter, shape, pore size and surface chemistry) of silica materials affect the immobilization and migration of labels, and the ultimate LFA performance. Such understandings may provide insights for the future development of LFAs with better sensitivity.

## 2. Colorimetric LFAs Using Silica Nanomaterials

Colorimetric LFAs are the most widely used strategy, as the generated signal can be directly read by the naked eye without using an additional device. With the accumulation of a large number of labels at the test line, a visible detection signal is formed [27]. Over the past few decades, gold nanoparticles (AuNPs) of various sizes have become the most used labels for colorimetric LFAs and are applicable for detection in different biofluids [9,29,30,31], mainly due to their excellent colloidal stability and characteristic red color [32]. However, the sensitivity of colorimetric LFAs is usually limited due to the brightness of the label [33]. Moreover, the surface charge of AuNPs is easily affected by the surrounding substance, such as a high-salt solution; this causes the aggregation of AuNPs, which influences their fluidity in LFA strips [34]. In addition, the sensitivity of the AuNP label is limited by the particle size. As the size of the AuNPs increases, they will show an intense color but decreased mobility. Several strategies have been developed to solve these problems, including the growth of metal shells [35,36,37], enzymatic deposition [38,39] and nanoparticle modification [40]; e.g., the use of silica nanomaterials is one of these strategies. Many researchers have reported the use of silica shells to protect AuNPs from aggregation [34]. In other cases, mesoporous silica nanoparticles have been utilized as carriers to load a large amount of AuNPs for signal amplification [41,42]. In addition, colored silica nanomaterials have also been developed as LFA labels by modifying silica nanomaterials with dye, showing satisfactory fluidity and bright colors [43].

**Figure 2:**
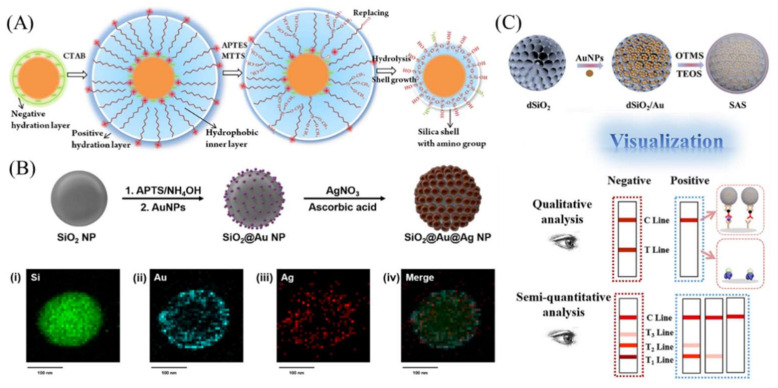
Silica-nanomaterial-assisted colorimetric LFA. (**A**) A silica shell was grown on AuNPs to enhance stability and avoid aggregation of label materials in order to enhance the detection sensitivity for vanillin in milk powder. (**B**) Multiple AuNPs were deposited onto solid silica sphere for the semiquantitative detection of prostate-specific antigens. (**C**) Multiple AuNPs were loaded into dendritic mesoporous silica nanoparticles for semiquantitative analysis. Reprinted with permission from References [21,34,41].

With the advantages of high stability in a high-salt environment and the ease of surface modification, silica nanomaterials have been used to protect AuNPs from aggregation and to enhance their binding efficiency to antibodies [44]. Lu et al. reported the synthesis of spherical core–shell gold–silica nanoparticles (AuNP@SiO_2_ NPs) as a label for the detection of alpha-fetoprotein (AFP) and vanillin (Figure 2A) [34]. With the protection of the SiO_2_ shell, AuNP@SiO_2_ NPs showed higher stability against the surrounding substances than bare AuNPs. With this design, the limit of detection (LOD) of AFP was enhanced by 30 times (300 pg mL^−1^).

Due to its relatively low sensitivity, a AuNP label is not suitable for the detection of low-abundance samples [45], which hinders its applications in the early diagnosis of diseases. Recent research shows that, when a number of AuNPs assemble together, the total light absorption greatly increases with improved signal intensity [46]. Due to their large specific surface area and the ease of surface modification, silica nanomaterials with various structures have been employed for use as carriers to enrich AuNPs. Dong et al. developed an LFA device by immobilizing a number of AuNPs in a solid silica sphere via electrostatic interaction for microRNA-21 detection [45]. This has been demonstrated to be a feasible method for the detection of miRNA-21 in cancer cells and human serum, with 60 times enhancement in sensitivity compared to that of single-AuNP-based LFAs. Kim et al. deposited Au-Ag alloy NPs on silica surfaces to enhance the signal intensity (Figure 2B). This assay was applied for early cancer screening by detecting blood prostate-specific antigen (PSA) levels in clinical samples [21]. It lowered the detection limit of PSA to 0.30 ng mL^−1^ without nonspecific binding, with a linear relationship in the PSA concentration range of 0–300 ng mL^−1^, which enabled a semiquantitative analysis.

Compared to solid nanoparticles, mesoporous silica nanoparticles (MSNs) with a larger surface area offer more capacity for label loading. Li et al. reported a novel competitive LFA for visual and semiquantitative analyses of enrofloxacin (ERN) residues [41]. The labels were prepared by loading AuNPs into the dendritic mesoporous silica, followed by silica encapsulation to prevent the leakage of the AuNPs (Figure 2C). This novel LFA offered an ultrasensitive detection of ERN, with detection limits of 0.125 ng mL^−1^ with the naked eye and 7.8 pg mL^−1^ with a scanner or smartphone, which are much lower than those of traditional gold and fluorescent LFA strips. Moreover, by designing a multi-range gradient LFA strip, they realized a visually semiquantitative identification of ENR. Xu et al. fabricated a gold-nanoparticle-decorated silica nanorod for use as an LFA label for ultrasensitive protein detection [47]. The assembly of AuNPs on a silica nanorod provided a purple color, darker than that of traditional AuNPs. The LOD of rabbit IgG was 0.01 ng mL^−1^, which is 50-fold lower than that of single-AuNP-based LFAs and comparable with that of fluorescent LFAs.

Some researchers have also doped silica nanomaterials with organic dyes for use as LFA labels. Compared with AuNPs, dye-doped silica nanomaterials have robust structures, a durable color, good chemical stability, and are cheap and easy to manufacture [43]. A series of dyed silica nanomaterials have been developed for use as LFA labels for biomarker detection. For example, purple silica nanomaterials were synthesized for the detection of clenbuterol [43] and showed a sensitivity comparable to that of AuNP-based LFAs. Later, red and blue silica nanomaterials were also fabricated for the detection of Escherichia coli O157:H7 [23] and hepatitis B surface antigens [48]. All studies indicated that colored silica nanomaterials are more tolerant to salt and extreme pH than AuNPs, suggesting that they are suitable for detection in various real samples. Nevertheless, the brightness of colored silica nanomaterials needs to be enhanced in order to improve their performance.

## 3. Fluorescent LFAs Using Silica Nanomaterials

Fluorescent LFAs utilize the characteristics of fluorescent probes, which absorb light energy of a specific wavelength and emit light of a longer wavelength [49] to identify biomarkers. Because fluorescent probes have a higher brightness than colorimetric probes, they have been reported to enhance LFA sensitivity by at least 10 times [50,51]. Their limitations are that an additional light source and a fluorescence reader are required.

### 3.1. Quantum Dots

Quantum dots (QDs) are semiconductor nanoparticles. Compared to bulk materials, QDs have unique optical and electronic properties. When excited by UV light, QDs emit fluorescent light dependent on their size and shape. QDs have been widely used in LFAs due to their high brightness and photostability [52]. However, most QDs are only soluble in organic solvents; thus, a hydrophilization step is required before applying QDs in LFAs [19].

**Figure 3:**
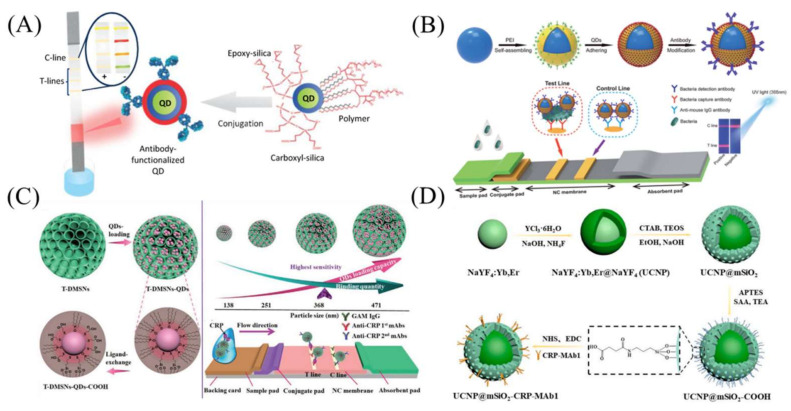
Silica-nanomaterial-assisted fluorescent LFA. (**A**) LFA using silica-shell-encapsulated QDs for simultaneous detection of four mycotoxins. (**B**) A core–shell SiO_2_-QD nanocomposite (SiO_2_@PEI-QD) was developed for use as an LFA label for rapid detection of Salmonella typhimurium in milk. (**C**) QDs were loaded into DMSNs for signal amplification, and the impacts of DMSN size on QD–DMSN interaction and the LOD of resultant LFA were also investigated. (**D**) Dendritic mesoporous silica-encapsulated UCNPs for SARS-CoV-2 detection. Reprinted with permission from References [6,19,56,65].

A simple strategy that can be used is to coat a silica shell in order to protect QDs and transfer QDs to aqueous media. The reason for choosing silica is because of its stability and easy modification properties. Additionally, a compact silica shell could prevent the possible release of toxic heavy metal ions from QDs. Foubert et al. developed a multiplex detection format LFA using silica-coated QDs (Figure 3A) [19]. The developed LFA with four QD label colors was used to detect four mycotoxins in one strip, and it achieved 100% specificity and over 95% sensitivity for all the mycotoxins. Later, Goryacheva et al. further enhanced the brightness of QDs by optimizing the silanization conditions of QDs [53]. They were applied for the detection of two mycotoxins in maize and wheat samples. The developed LFA was validated by comparing its accuracy to that of liquid chromatography coupled to tandem mass spectrometry (LC-MS/MS).

Another strategy is the use of silica nanomaterials as a hydrophilic supporting core to immobilize QDs and provide good dispersity. Zhang et al. synthesized a core–shell SiO_2_-QD nanocomposite (SiO_2_@PEI-QDs) for use as an LFA label for the rapid detection of Salmonella typhimurium in milk (Figure 3B) [6]. Compared with commercial bare QD labels, SiO_2_@PEI-QDs exhibited higher dispersity and uniformity, and they enabled a 20 times lower LOD. Wang et al. reported the use of a novel silica-core@dual QD-shell nanocomposite (SiO_2_@DQD) as an LFA label for the simple and sensitive detection of SARS-CoV-2-specific antibodies: immunoglobulin-M (IgM) and immunoglobulin-G (IgG) [54]. The clinical practicality of the developed LFA was evaluated by testing 361 clinical specimens. The results showed that both the sensitivity and specificity were above 95%, with the sensitivity being higher than that of AuNP-based LFAs (88.66% sensitivity).

Compared to solid silica cores, dendritic mesoporous silica nanoparticles (DMSNs) with large and open pores have been applied to enable higher QD loading for better detection sensitivity [55]. Huang et al. synthesized DMSN-based pitaya-type dSiO_2_/QDs/SiO_2_ spheres (SQSs) for use as LFA labels for C-reactive protein (CRP) detection [55]. The QDs were loaded into the porous structure and further encapsulated with a silica shell to achieve high brightness and hydrophilicity. The resultant SQSs exhibited a QD loading amount ten times higher than that of non-porous nanospheres, which contributed to a lower LOD for CRP. Recently, Gao et al. reported a ligand exchange strategy to transfer hydrophobic QDs into an aqueous phase [56]. Compared to the silica encapsulation method, this strategy is simple and causes few surface defects on QDs, which is beneficial for the fluorescence preservation of QDs for a low LOD. The impacts of DMSN size [57], surface chemistry [56] and QD size [58] on QD–DMSN interaction and the resultant LFA LOD were systematically investigated in Gao’s studies. A series of DMSNs with various amino and thiol densities were prepared for QD loading as LFA labels [56]. It was demonstrated that a high thiol density resulted in high QD loading, and the amino groups contributed to QD fluorescence preservation. The optimized LFA labels with an amino density of 153 μmol g^−1^ and a thiol density of 218 μmol g^−1^ enabled a sensitive detection of serum amyloid A (SAA) at 10 pg mL^−1^, which is one order of magnitude lower than that reported in the literature. The influence of DMSN size was studied in a size range of 100–500 nm, and the best LOD of CRP (10 pg mL^−1^) was achieved with a size of 368 nm (Figure 3C) [57]. Furthermore, they also optimized the QD size to balance the loading amount and quantum yields [58], and the LOD of CPR was further lowered to 5 pg mL^−1^. Liang et al. loaded both QDs and iron oxide nanoparticles into DMSNs for procalcitonin detection [59]. Here, iron oxide nanoparticles were used for magnetic separation. Fluorescent carbon dots have also been used as LFA labels [60] because they have better chemical inertness, photostability, biocompatibility and lower cytotoxicity than conventional QDs. Xu et al. synthesized a new type of fluorescent carbon-dots-based SiO_2_ sphere as an LFA label for the detection of the Zika virus in patients’ sera [60]. The results showed that the LOD of this type of LFA was 100-fold lower than that of traditional AuNP-based LFAs.

### 3.2. Other Fluorescent Materials Using Silica Nanomaterials

In addition to QDs, silica nanomaterials have also been loaded with other fluorescent materials, such as upconversion nanoparticles (UCNPs) and fluorescent dyes [49]. UCNPs are types of rare-earth-element-doped luminescent nanomaterials, which convert low-energy radiation into high-energy radiation [61]. With less interference from biological autofluorescence, UCNPs are good label materials for LFAs [62]. However, most UCNPs are synthesized in non-aqueous solutions with poor water dispersibility [62]; thus, they require surface modification to improve their hydrophilicity [63,64]. Guo et al. reported the synthesis of mesoporous silica-encapsulated UCNPs (UCNPs@mSiO_2_) for use as fluorescent labels for SARS-CoV-2 detection (Figure 3D) [65]. The mesoporous silica shell enhanced the aqueous dispersibility of UCNPs and provided a reactive surface for antibody immobilization. This LFA was fabricated into a 5G-enabled device to realize the real-time recording of patient data. Wiriyachaiporn et al. reported a simple fluorescent-based LFA platform using Cy5-dye-doped silica nanomaterials as fluorescent labels for rapid influenza B virus screening [66]. This work suggested that the carboxyl modification of silica nanomaterials could prevent the denaturation of biomolecules in harsh environments.

### 3.3. Dual-Model LFA Using Silica Nanomaterials

**Figure 4:**
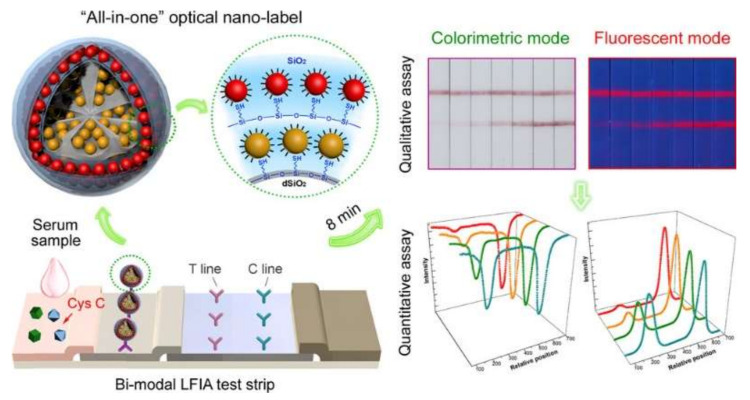
Dual-model LFA with both QDs (florescent) and AuNPs (colorimetric) integrated into DMSNs for early diagnosis of kidney injury. Reprinted with permission from Reference [22].

LFAs with dual-signal labels that realize colorimetric and fluorescent detection at the same time have also been reported [22]. As shown in Figure 4, DMSNs with a large internal space were utilized to firstly load AuNPs through thiol–metal coordination and then densely pack them with hydrophobic QDs, followed by silica encapsulation. This novel LFA strip was used for the rapid and sensitive screening of Cys C, which is important for the early diagnosis of kidney injury. Taking advantage of the enhanced colorimetric and fluorescent signals of the labels, these novel LFA strips realized dual-signal qualitative sensing and a quantitative analysis of Cys C within 8 min. Preechakasedkit et al. also developed a dual-model LFA by using hybrid nanocomposite materials (AuNPs@SiO_2_-Eu^3+^) as the label [20]. A silica layer was created to protect the fluorescent Eu^3+^ from environmental influence (e.g., photo-decomposition) and quenching by the Au core, resulting in a stability higher than that of free fluorophores. This assay enables the sensitive detection of human thyroid-stimulating hormone levels.

## 4. Chemiluminescent LFAs (CL-LFAs) Using Silica Nanomaterials

Chemiluminescence is the emission of light produced by a chemical reaction. Compared with fluorescent LFAs, CL-LFAs have a simple setup because they do not require an excitation light source or a fluorescence detector, thus resulting in lower background noise [67]. A well-known chemiluminescence example is horseradish peroxidase (HRP); this oxidizes luminol to produce colored 3-aminophthalate, which is widely used in commercial ELISA kits. Jung et al. synthesized HRP-loaded porous silica nanoparticles with large cavities for use as CL-LFA labels for the rapid and sensitive detection of avian influenza viruses (AIV) [68]. The suitable pore size of porous silica nanoparticles ensured that HRP was loaded into the pores to achieve a high loading amount, while the antibodies with a large molecular size were located on the outer surface to enhance accessibility to the antigen. When this CL-LFA was applied for AVI detection in clinical specimens, a 20-fold enhancement in the LOD was achieved compared with that of commercial kits. In another study, a CL-LFA was developed using dye-doped porous silica nanomaterials and TCPO (bis(2,4,6-trichlorophenyl) oxalate, H_2_O_2_ and imidazole) [69]. This LFA strip was used for the detection of staphylococcal enterotoxin C1, which is an important pathogenic substance that causes food poisoning. The achieved LOD was an order of magnitude lower than that of the ELISA method. However, the authors mentioned that this chemiluminescence method has a limited linear range and that the stability/storage of the label materials should be improved.

**Figure 5:**
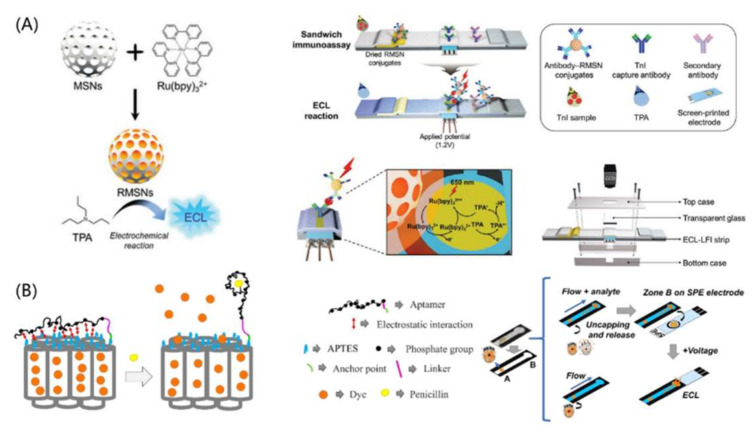
Silica-nanomaterial-assisted ECL-LFA. (**A**) [Ru(Bpy)_3_]^2+^-loaded MSNs as ECL probes for sensitive and quantitative detection of troponin I. (**B**) MSN-based aptamer-gated indicator releasing strategy for antibiotic detection. Reprinted with permission from References [70,72].

Electrochemiluminescent LFAs (ECL-LFAs) are another type of CL-LFAs, the luminescent signal of which is also produced by a chemical reaction, while the chemical reaction is initiated by an electrical potential and occurs at the electrode [70]. Ruthenium complexes, especially [Ru(Bpy)_3_]^2+^, are widely used for ECL-LFAs [71,72,73]. In the presence of tripropylamine (TPrA), [Ru(Bpy)_3_]^2+^ can be excited and emit luminescence at ~650 nm upon relaxation [25]. In addition to low background noise, ECL-LFAs have another advantage of a controllable reaction process, which is realized by applying a voltage to the electrodes. MSNs have been shown to be an ideal carrier for [Ru(Bpy)_3_]^2+^ and have an enhanced ECL signal due to their high loading capacity [74,75]. Compared to nonporous nanostructures, MSNs with high specific surface areas and adjustable pore sizes were found to exhibit a higher [Ru(Bpy)_3_]^2+^ loading capacity and outstanding ECL efficiency [72,76]. [Ru(Bpy)_3_]^2+^-loaded MSNs (RMSNs) were also used as an ECL-LFA label for the detection of troponin I (TnI) (Figure 5A) [72], and they achieved a better LOD (three orders of magnitude) than fluorescent LFAs. Additionally, the quantification of TnI in clinical samples was demonstrated in this study. Climent et al. combined ECL detection with an aptamer-gated indicator releasing strategy for the rapid detection of penicillin in milk [70]. In the presence of the target analyte, the gatekeeper aptamer will bind with the analyte and release the dye loaded in the pores of MSNs (Figure 5B). Compared with conventional methods, this approach presents advantages when analyzing challenging matrices and is suitable for ultra-trace analysis.

## 5. Surface-Enhanced Raman Scattering LFAs (SERS-LFAs)

Surface-enhanced Raman scattering (SERS) is an ultrasensitive detection method. It enhances Raman scattering by molecules adsorbed on rough metal surfaces or nanostructures [77]. The most common method for performing SERS measurements is depositing samples onto a silicon or glass surface with a nanostructured noble metal surface [78]. The enhancement factor can be as much as 10^10^ to 10^11^. Thus, it is an ultrasensitive method that enables the detection of a single molecule. Recently, gold nanosphere-based SERS labels have been shown to have high sensitivity and strong anti-interference ability; thus, they are widely used in LFA measurements. Many silica nanostructures have also been used to further improve the stability of SERS labels.

Gao et al. used an SERS-LFA for the detection of neuron-specific enolase (NSE) in blood plasma [79]. NSE is an important biomarker for the diagnosis of traumatic brain injury. Traditional detection methods, such as ELISA and Western blot, are time-consuming and require large sample volumes and expensive instruments. By synthesizing Au nanostar@Raman Reporter@silica sandwich nanoparticles, researchers successfully solved the instability issue of bare AuNPs, and silica nanomaterials shells could also prevent the leakage of Raman reporters. The LOD of this SERS-LFA was three orders of magnitude lower than that of colorimetric LFAs. Later, these novel nanoparticles were used for the detection of carcinoembryonic antigens (CEAs) in a drop of whole blood [80]. Combining three layers of filter membranes with an LFA strip to realize the separation of plasma, the LOD was lowered to 1.0 ng mL^−1^.

**Figure 6:**
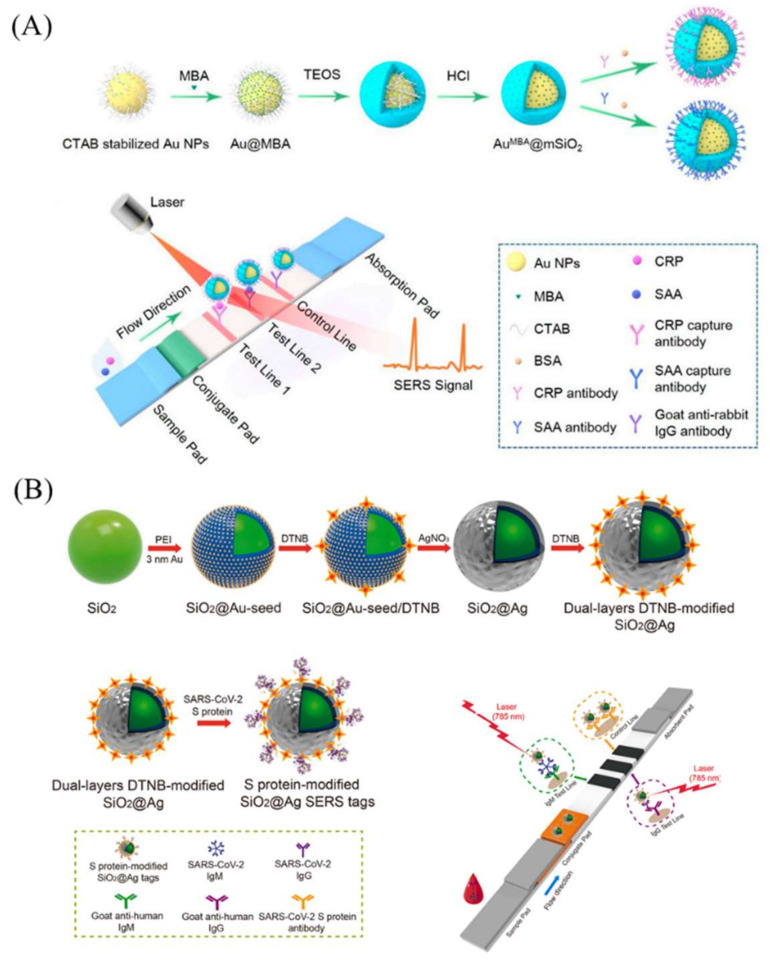
Silica-nanomaterial-assisted SERS-LFA. (**A**) Core–shell Au/MBA@mSiO_2_ nanoparticles used as SERS-LFA labels for co-detection of CRP and SAA. (**B**) Silica sphere immobilized dual-layer Raman reporters for anti-SARS-CoV-2 IgM/IgG detection. Reprinted with permission from References [81,84].

Li et al. synthesized core–shell Au/MBA@mSiO_2_ nanoparticles for use as SERS-LFA labels [81]. The AuNPs were encapsulated by mesoporous silica; meanwhile, the Raman reporter mercaptobenzoic acid was loaded in the pores of mSiO_2_ (Figure 6A). This design was used for the detection of two infection biomarkers (SAA and CRP) at the same time. The detection limits of SAA and CRP were 0.1 and 0.05 ng mL^−1^, respectively, both of which were ~10 times more sensitive than those reported in the literature. Additionally, the SERS-LFA strips could be analyzed using a smartphone-based portable Raman spectrometer, which is useful in remote areas without enough medical resources.

Jeon et al. also fabricated silica-encapsulated AuNPs for use as SERS-LFA labels with improved stability and reproducibility [82]. Raman reporter molecules could be dissociated from the surface of the AuNPs when exposed to high temperatures, extreme pH or high-salt conditions [83,84]. In this study, the silica shell protected AuNPs from the influences of the harsh environment, making them suitable for the detection of mosquito-borne diseases in tropical regions.

Liu et al. developed a SiO_2_@Ag nanocomposite for use as high-performance SERS tags for the rapid and ultrasensitive detection of anti-SARS-CoV-2 IgM/IgG in clinical samples (Figure 6B) [84]. The 200 nm silica nanomaterial core worked as a highly stable and monodispersed supporter for Raman reporters’ immobilization. The resultant LOD of this method was 800 times lower than that of a commercial colloid gold strip kit.

## 6. Summary and Outlook

As rapid point-of-care diagnostic devices, LFAs have become the key technology in the control of infectious diseases such as COVID-19. With the advancement of nanotechnology, many silica nanomaterials with unique properties, such as a large specific surface area, a controllable structure and the ease of surface modification, have been applied to immobilize various labels in order to enhance LFA sensitivity. Silica-nanomaterial-enriched labels have shown enhanced signal intensity, improved hydrophilicity and better tolerance to salt and pH. In addition, dual-mode detection has also been achieved by using porous silica nanomaterials to carry multiple types of label tags.

Despite the recent progress, there is plenty of room for improvement in silica-nanomaterial-assisted LFAs. One of the future research directions is multiplex quantitative detection. Multiplex quantitative detection enables the detection of several target molecules in a single strip, which not only shortens the detection time but also reduces the cost. Additionally, reducing the size of the reader instrument is also an important development direction for the wide use of LFAs in rural areas. Cloud-enabled smartphones with an implanted reader have a promising future, because they can quickly capture and analyze images of the results and upload the latest information concerning patients’ conditions to the cloud. Last but not least, the quality control of silica nanomaterials used for the immobilization of labels is also crucial to ensure reproducibility. It is expected that more innovative LFAs will be developed in the near future. Their commercialization will benefit the whole of society.
